# Different LED light spectra’s and nano-chelated potassium affect the quality traits of Dolce Vita cut roses in soilless culture condition

**DOI:** 10.1038/s41598-023-34056-4

**Published:** 2023-04-25

**Authors:** Zahra Heidari, Parviz Noruzi, Javad Rezapour-fard, Zohreh Jabbarzadeh

**Affiliations:** Department of Horticultural Sciences, Faculty of Agriculture, Urmia University, P.O. Box: 165-5715944931, Urmia, Iran

**Keywords:** Light responses, Photosynthesis, Stomata, Plant physiology

## Abstract

Roses are classified as neutral day plants, but high light and cool temperatures produce high quality flowers in roses. As light quantity, the light quality and its special spectra can affect the flower yield and quality. This research aimed to study of the effect of LED light (control (sunlight), blue and red spectra’s) and nano-chelated potassium at three levels (0, 1.5 and 3 g/l) on some morphophysiological and biochemical traits of *Rosa hybrida* cv. Dolce Vita. Light and nano-chelated potassium treatments have a significant effect on most traits measured in the present study. According to the results, the use of red light and nano-chelated potassium in rose cultivation improved the quality characteristics and increased vase life. The highest fresh and dry weight of flowering branch and plant height was observed in red light treatment and the concentration of 3 g/l nano-chelated potassium. Biochemical parameters such as phenolic compounds, leaf and petal flavonoids, petal anthocyanin content, antioxidant capacity and vase life were also significantly increased under red light and with the concentration of 3 g/l nano-chelated potassium compared to the control. In general, it can be said that the use of red light and a concentration of 3 g/l nano-chelated potassium, can be effective in improving the quality of rose flowers, especially in low light condition.

## Introduction

The rose (*Rosa* × *hybrida* L.) belongs to the Rosaceae family and is one of the most popular flowers in the world. Roses are usually grown as cut flowers. Adequate light is one of the most influential factors in the production of high-quality rose flowers^1^. Plants use light as an energy source to carbon absorption in photosynthesis and to activate and regulate many physiological processes related to plant growth and development^2^. Light is not only an energy source for photosynthesis, but also a stimulus for a number of developmental processes from seed germination to the beginning of flowering^3^. Both the quality and quantity of incoming light can cause to severe effects on photosynthetic activity and the adaptation of the photosystem to changes in light quality^4^. Of the spectral distribution of solar radiation, only about 50% of the radiation that reaches the earth surface is photosynthetically active radiation (PAR). In sunlight, red and blue wavelengths are more important for photosynthesis. Other wavelengths, such as ultraviolet and far red, which are absorbed by certain light receptors, act as signal inducers for different developmental pathways. The use of artificial light is a common method of replacing or compensating daylight duration and intensity shortage for the growth of a variety of plant species in greenhouse crop production^5,6^. Greenhouse lighting is the most important parameter in plant production in the dim months of winter. Complementary light increases the rate of leaf photosynthesis and plant growth and quality^7^. Nowadays, modern greenhouses use supplementary light to increase the growth and production of agricultural products^8–10^. One of the most common complementary light sources are LEDs. LED lighting technology offers advantages such as long life, small volume, less heat dissipation, adjustable light intensity, high efficiency and the ability to specific wavelengths emitting^9,11^. In addition to light, plant nutrition has a key role in greenhouse crop production. Today, nano-fertilizers (NFs) opens a new horizon in fertilizer industry, which resulted in more nutrients efficiency for optimal crop production^12^. Nanoparticles (NPs) are small molecules with a small size range of 1–100 nm with different physicochemical properties than bulk materials. Nanoparticles improve physical, chemical, and biological properties and functions of source materials by increasing the surface-to-volume ratio^13^. The use of nano-fertilizers leads to increasing the nutrient utilization efficiency, reducing soil toxicity, alleviation the negative effects of excessive fertilizer application and better nutrient management. By using nano-fertilizers, the time and speed of nutrient releasing is in accordance with the nutritional needs of the plant, so the absorption of the nutrients well done, therefore, reduced element leaching and increased product performance are achievable^14^.

One of the most widely used nano-fertilizers in agriculture is potassium nano-chelates. Potassium is the most abundant cation in plant cells. Potassium ions (K^+^) act as highly mobile osmolytes that form weak complexes and remain easily interchangeable. K is mainly present in soluble form in the cellular cytosol^15^, and has a key role in the biosynthesis of anthocyanins. Potassium increases plant productivity and quality by enhancing the photosynthesis and formation of starch and proteins as well as ATP production, and activating at least 60 enzymes that are effective in growth. It also increases root growth and stress tolerance, helps better transportation of sugars produced in the plant, maintains cell turgidity, prevents the spread of plant diseases and nematodes, and regulates the stomatal opening and closing^16^. Dolce Vita rose have a predominant white flower color with pink edges. The optimal color appearance of the petals edge is closely related to the quantity and quality of light and plays a determinative role in the commercial value of this cultivar. One of the problems in growing this rose cultivar, especially in low light seasons, is that the flowers often turn white and the pink color of the petals margin disappears. This complication reduces the quality and marketability of the product. To intense the flower edge color and improve the quality and floral display of cut Dolce Vita rose flowers, this research was done. According to the mentioned cases, the effect of blue and red spectra from LED lighting source as supplementary light and foliar application of nano-chelated potassium and their interactions on anthocyanin content and some morphophysiological and biochemical properties of Dolce Vita rose cultivar was studied under soilless cultivation.

## Materials and methods

### Plant material and experimental design

This research was conducted in the greenhouse unit and research laboratories of the horticulture department of Urmia University, Urmia, Iran. Newly grafted rose seedlings of Dolce Vita cultivar were purchased from a local commercial nursery and each seedling was planted in a 10 L plastic pot containing peat moss and perlite in a ratio of 75 to 25 percent. The pots were transferred under drip irrigation system and plants fertigated with a modified commercial rose fertilizing recipe using a pulse irrigation strategy five times a day. The potassium content of used recipe reduced to half strength and final nutrient solution contained 150 mg/l N, 45 mg/l P, 125 mg/l K, 120 mg/l Ca, 40 mg/l Mg and also trace elements. More details showed in Table 1.

**Table 1:** Fertilizer type and amount used to make 1000 L of nutrient solution.

Fertilizer type	Amount (g/1000 l)	N (mg/l)		P (mg/l)	K (mg/l)	Ca (mg/l)	Mg (mg/l)
		N-NO3	N-NH4				
Monoammonium phosphate (NH_4_H_2_PO_4_)	169	–	20	45	–	–	–
Calcium nitrate (5Ca(NO_3_)_2_-NH_4_NO_3_.10H_2_O), Agricultural grade	631	91	7	–	–	120	–
Potassium nitrate (KNO_3_)	246	32	–	–	93.5	–	–
Magnesium sulfate (MgSO_4_.7H_2_O)	440	–	–	–	–	–	40
Potassium sulfate (K_2_SO_4_)	73	–	–	–	31.5	–	–
Total elemental concentration (mg/l)		150		45	125	120	40

**Figure 1:**
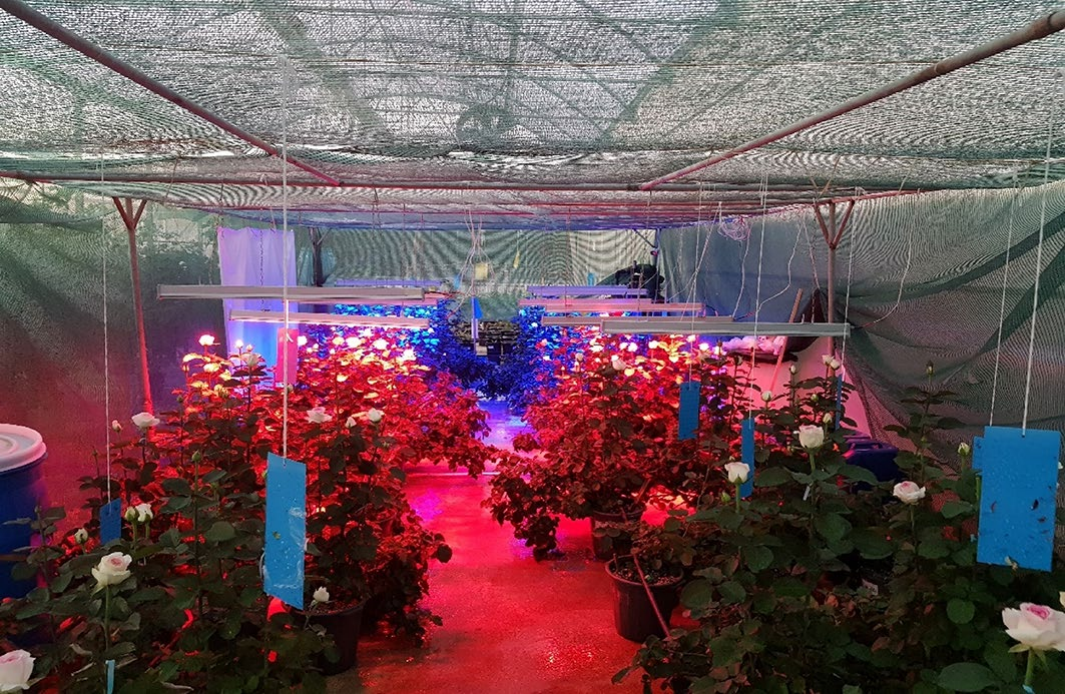
Treatment of Dolce Vita roses with blue and red light.

Experimental treatments included foliar application with nano-chelated potassium 27% at three levels (0, 1.5 and 3 g/l) and light at three levels (control (sunlight), blue and red spectra’s) as supplementary light that was performed as factorial in a completely randomized design with six replications. The nano-chelated potassium prepared by the method described by Nido et al.^17^. For this, a four step process ran including the preparation of potassium alginate solution, pre-gelation, stabilization and equilibration. For the preparation of potassium alginate solution (K-ALG), 117.5 ml of alginate stock solution (6 mM sodium alginate, pH 4.9) was mixed with KCl (as potassium source) and sonicated in Elmasonic P 120 H sonicator for 20 min. To form pre-gel, 36 mM calcium chloride solution (pH 7) was added drop by drop to K-ALG while sonicating at 25 °C. On stabilization step, 25 ml of chitosan stock solution (CHI), prepared by dissolving 0.10 mg/ml low molecular weight chitosan in 1% acetic acid, was added dropwise to K-ALG-CHI mixture while stirring for 30 min. For chemical reaction completion, the prepared mixture was kept for 24 h at room temperature and finally, the solution was oven-dried at 75 °C for 1 h to obtain solid fertilizer. The potassium content assayed by Jenway PFP7 flame photometer. Chemical materials used in this method, were purchased from Sigma-Aldrich chemicals Company. The application of nano-chelated potassium and lighting treatments started just one week after first stem bending and when the new shoots reached to 3–4 cm height. Nano-chelated potassium used once a week as foliar application. The using of red and blue light as supplementary light started simultaneously with the application of nano-chelated potassium. Supplementary LED lighting as red and blue light used with the intensity of 95 µmol m^−2^ s^−1^ (Fig. 1). Total light intensity was about 530 µmol m^−2^ s^−1^ at the growing point of plants. Day/night temperature was adjusted to 27/22 °C during growing period and the relative humidity maintained at 55%. Plant samples were taken to evaluate the morphophysiological and biochemical characteristics.

### Morphological traits

After harvesting the flowering stem, the stem length from the cut point to the tip of the bud was measured and reported in centimeters. In order to determine the leaf area, three fully developed leaves were randomly selected from shoot middle and the leaf area was measured by a leaf area meter (AM 200). The fresh weight of the whole flower stems was measured by a digital scale with an accuracy of 0.01 g. To determine the dry weight of the stem, they were placed separately in a paper bag in a 70 °C oven for 72 h and then measured by a digital scale with an accuracy of 0.001 g. Leaf thickness was measured by a high resolution micrometer (Mitutoyo Digital Micrometer, Model 293-185. Kawasaki, Japan).

### Flower vase life

The well-developed flower stems were harvested in commercial stage and flower longevity was assessed based on method described by Aghdam et al.^18^. Light intensity of storage room adjusted to 60 µmol m^−2^ s^−1^ that supplied by a mixture of warm and cold white inflorescent lamps. Stem recut was done every three days.

### Petal anthocyanin

The anthocyanin content of petals in all extracts was measured by spectrophotometer and pH-differential method^19^ using two buffer systems, potassium chloride buffer (0.025 M, pH = 1) and sodium acetate buffer (0.4 M, pH = 4.5). The results were expressed in milligrams per liter equivalent to cyanidin-3-glycoside in the extract based on the following formula:

The density of monomeric anthocyanin pigment in the extract in mg/l is equal to (Ɛ × 1) (A × MW × DF × 1000), in which DF, MW, Ɛ and A refer to sample dilution factor, Molecular weight of cyanidin, Molar coefficient of cyanidin, and the amount of adsorption, respectively. The amount of absorption is calculated as follows:

$${\text{A}} = \left( {{\text{A}}_{{{51}0}} - {\text{A}}_{{{7}00}} } \right){\text{ pH 1}}.0 - \left( {{\text{A}}_{{{51}0}} - {\text{A}}_{{{7}00}} } \right){\text{ pH 4}}.{5}$$

The A_510_ and A_700_ represent absorbance of 510 nm and 700 nm, respectively.

### Chlorophyll content

Photosynthetic pigments measurement was done using Gross^20^ method and the absorbance was read at 645 and 663 nm using a spectrophotometer (UV/Visible Lambda 25 Perkin Elmer). The amount of chlorophylls a, b and total were calculated from the following equations and reported in mg/g fresh weight.

1 $${\text{chlorophyll a }}\left( {{\text{g}}/{\text{l}}} \right) = \left( {0.0{127} \times {\text{OD}}_{{{663}}} } \right) - \left( {0.00{269} \times {\text{OD}}_{{{645}}} } \right)$$

2 $${\text{chlorophyll b }}\left( {{\text{g}}/{\text{l}}} \right) = \left( {0.0{229} \times {\text{OD}}_{{{645}}} } \right) - \left( {0.00{468} \times {\text{OD}}_{{{663}}} } \right)$$

3 $${\text{Total chlorophyll }}\left( {{\text{g}}/{\text{l}}} \right) = \left( {0.0{2}0{2} \times {\text{OD}}_{{{645}}} } \right) + \left( {0.00{8}0{2} \times {\text{OD}}_{{{663}}} } \right)$$

In the above relations, OD_663_ and OD_645_ are the absorption rates at 663 and 645 nm, respectively.

### Antioxidant capacity

DPPH method was used to measure antioxidant capacity. To measure the ability of extracts to inhibit free radicals (DPPH), first 100 μl of methanolic extract was mixed with 1900 μl of DPPH and then the samples were placed in the dark at room temperature for 30 min. Then, the absorbance of the samples was read by spectrophotometer at 517 nm and expressed by the percentage of inhibition^21^.

$${\text{Inhibition percentage}} = \left( {{\text{Ac}} - {\text{As}}} \right)/{\text{Ac}}*{1}00\%$$

Ac = control absorption.

As = sample absorption.

### Stomata conductance and Photosynthesis rate

Leaf porometer (SC-1, Meter group, Inc. USA) was used to determine the stomatal conductance. The rate of photosynthesis measured by a portable photosynthesis system (HCM-1000, Heinz Walz GmbH. Germany. Probe model: 1050-H).

### Petal phenolics

Total phenolic content was measured using Folin-Ciocalteu reagent method described by Marinova et al.^22^ with some minor modifications in weight of plant sample and dilution factor. The absorbance of the samples was read at 750 nm. The standard curve of Gallic acid was used to calculate the amount of total phenol content and the amount of phenol was calculated based on the standard curve of Gallic acid using the following equation in milligrams of Gallic acid per gram of fresh weight.

$${\text{Y}} = \, 0.0{\text{669x}} + 0.0{116}$$

Y = a number read on a spectrophotometer.

X = amount of phenol in mg/g fresh weight.

### Flavonoids content of leaves and petals

Aluminum chloride colorimetric method^23^ was used to measure flavonoids content in leaves and petals. The absorbance of the samples was read at 415 nm. The total flavonoids content of the samples was expressed in mg equivalent to quercetin per gram of plant dry weight.

### Petal H_2_O_2_ content

The amount of H_2_O_2_ measured using a method described by Velikova et al.^24^. The sample absorbance was read at 390 nm and a standard curve was used to calculate the hydrogen peroxide concentration and the results were presented in micromoles per gram of fresh weight.

### Leaf protein content

Bradford method^25^ was used to determine the amount of soluble proteins. The reading was performed with a spectrophotometer at 585 nm. The amount of protein was calculated after drawing the protein standard curve and was expressed in milligrams per gram of fresh weight using the following equation.

$$y = { 2}.0{36}x + \, 0.0{16}$$

*y* is the value read in the spectrophotometer and *x* is the value to be calculated.

### Carbohydrate content

0.5 g of leaf tissue was used to measure dissolved sugars according to Irigoyen et al.^26^ method. To drawing the glucose standard curve, solutions of glucose with concentrations of 0 to 120 mg/l were prepared and all experimental steps were performed on them, and finally the absorbance was read at 625 nm.

### Ethical approval

Authors confirm that all methods used in this study were performed in accordance with the relevant international and/or institutional guidelines and regulations.

## Results

### Flower stem height

**Figure 2:**
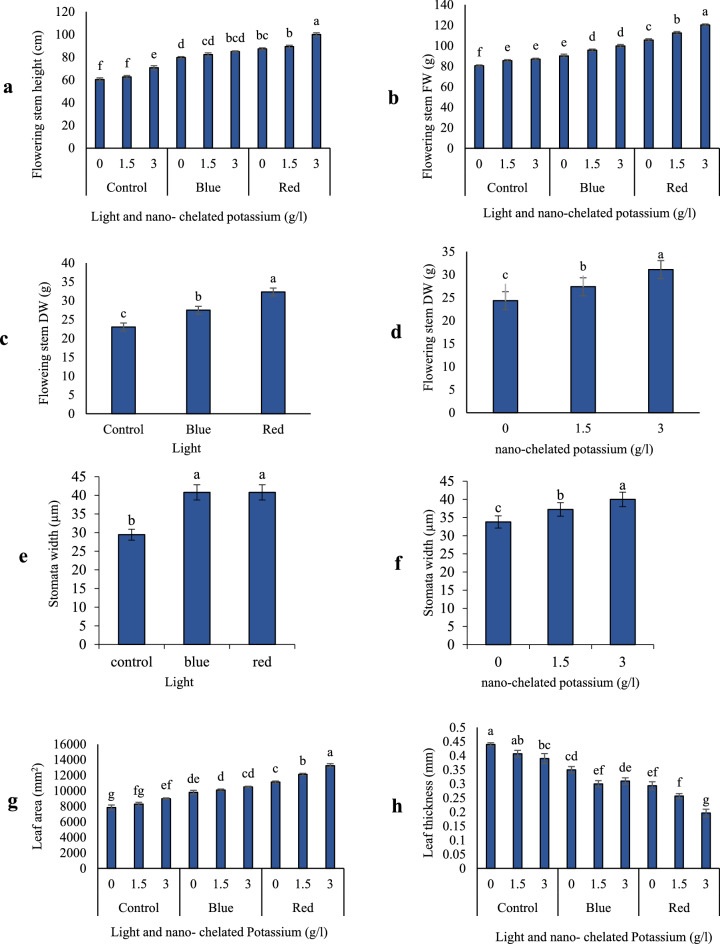
The biomass and some morphological traits of Dolce Vita rose in response to the different light and nano-chelated potassium levels: (**a**) flowering stem height, (**b**) flowering stem FW, (**c**,**d**) Flowering stem DW, (**e**,**f**) stomata width, (**g**) leaf area and (**h**) leaf thickness. *FW* fresh weight, *DW* dry weight.

Based on ANOVA the interaction effect of light and nano-chelated potassium was significant on flower stem height (Table 2). As shown in Fig. 2a, the highest flower stem height (100 cm) was observed in the red light treatment and a concentration of 3 g/l nano-chelated potassium.

**Table 2:** Results of ANOVA table for measured traits due to light and nano-chelated potassium treatments.

S.O.V	df	Critical *F* value	*F* values																	
			Flowering shoot height	Flowering shoot FW	Flowering shoot DW	Stomata width	Leaf area	Leaf thickness	Stomatal conductance	Total chlorophyll	Photosynthesis	Carbohydrate	Total protein	Petal anthocyanin	Total phenol	Leaf flavonoids	Petal flavonoid	Antioxidant capacity	Hydrogen peroxide	Vase life
Light	2	6.01 (α = 0.01) 3.55 (α = 0.05)	367.39**	442.31**	168.35**	231.20**	267.10**	131.23**	5840.73**	54.69**	176.22**	1158.60**	542.24**	366.14**	4770.91**	537.08**	56.14**	749.60**	22.49**	298.09**
Nano-K	2	6.01 (α = 0.01) 3.55 (α = 0.05)	45.24**	57.12**	74.21**	52.47**	32.32**	17.63**	391.03**	19.19**	38.73**	71.10**	66.61**	39.20**	276.99**	52.66**	17.46**	104.46**	0.37^ns^	103.91**
Light × Nano-K	4	4.57 (α = 0.01) 2.92 (α = 0.05)	3.19*	3.31*	0.04^ns^	0.67^ns^	3.22*	3.28*	11.81**	11.78**	3.62*	0.89^ns^	16.49**	3.24*	49.96**	6.29**	5.36**	6.13**	0.73^ns^	3.55*

ns, *, **: Non-significant and significant at the probability level of 5% and 1%, respectively.

### Flower stem FW and DW

The interaction of light and nano-chelated potassium treatments caused an increase in the fresh weight of the flower stem. The highest fresh weight of flower stem (120.31 g) was observed in red light treatment and concentration of 3 g/l nano-chelated potassium, which significantly increased in compared to the control (80.48 g) (Fig. 2b). Light treatment increased the dry weight of flower stem that red light treatment had a greater effect (32.32 g) on increasing dry weight of flower stem. The lowest dry weight of flower stem (23.01 g) was observed in the control (Fig. 2c). According to Fig. 2d, it was found that nano-chelated potassium treatment increased the dry weight of flower stems. The concentration of 3 g/l nano-chelated potassium had the greatest effect on increasing the dry weight of flower stems.

### Stomata width

Based on our findings, light treatment increased the stomata width and as shown in Fig. 2e, the treatment of red and blue light on the stomata width was not statistically significant, but they had a significant difference with the control (29.44 µm). Increasing the concentration of nano-chelated potassium increased the stomata width so that the concentration of 3 g/l caused the highest stomata width (40 µm) which increased in compared to the control (Fig. 2f).

### Leaf area

According to the results from the ANOVA, it was found that the interaction of light treatment and nano-chelated potassium caused an increasing trend in leaf area. According to Fig. 2g, the highest leaf area (13,240.5 mm^2^) was observed in red light in combination with the concentration of 3 g/l nano-chelated potassium that resulted in a 1.68 times increase in leaf area compared to the control.

### Leaf thickness

The results showed that the combination of light and nano-chelated potassium treatments reduced leaf thickness in treated plants. As shown in Fig. 2h, the highest leaf thickness (0.440 mm) was observed in the control treatment and the lowest leaf thickness (0.196 mm) was observed in red light treatment with 3 g/l nano-chelated potassium.

### Stomatal conductance

**Figure 3:**
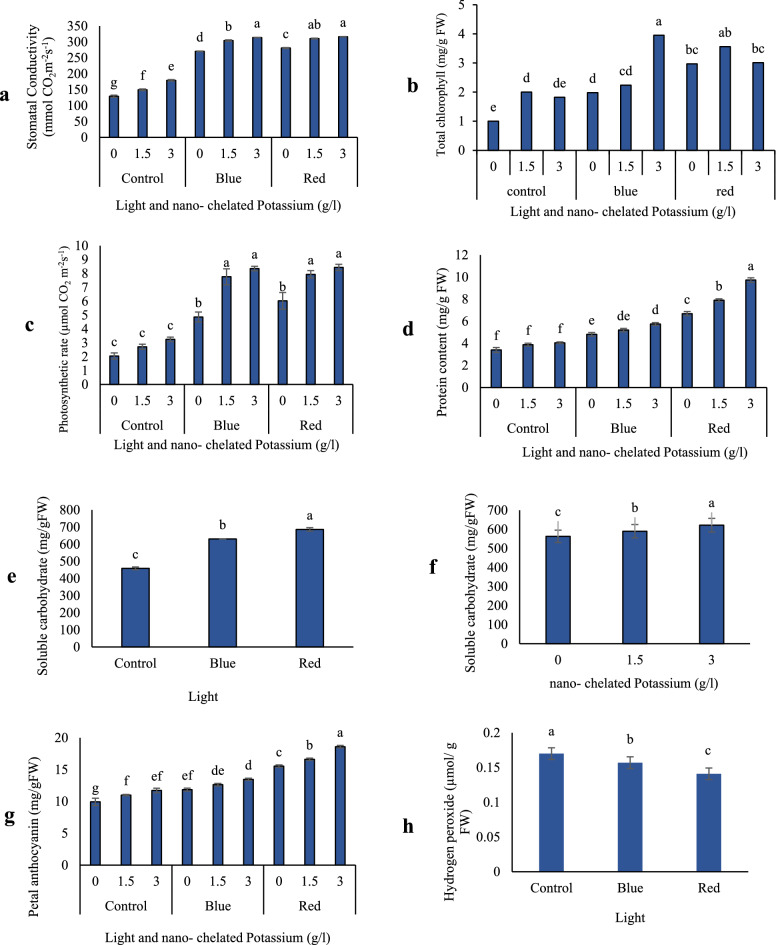
The physiological traits of Dolce Vita rose in response to the different light and nano-chelated potassium levels: (**a**) stomatal condactivity, (**b**) total chlorophyll, (**c**) photosynthesis rate, (**d**) protein content, (**e**,**f**) soluble carbohydrates, (**g**) petal anthocyanin and (**h**) H_2_O_2_ content. *FW* fresh weight, *DW* dry weight.

The interactions of light and nano-chelated potassium has a significant effect on stomatal conductance at 1% probability level (Table 2). Results showed that light and nano-chelated potassium treatment increased the stomatal conductance so that the highest stomatal conductance (316.53 mmol CO_2_ m^−2^ s^−1^) was observed in red light treatment and concentration of 3 g/l nano-chelated potassium, Although there was no statistically significant difference between red light treatments with concentration of 1.5 g/l and blue light treatments with concentration of 3 g/l thousand nano-chelated potassium. The lowest stomatal conductance was observed in the control treatment. As Fig. 3a shows, the treatment of red light and nano-chelated potassium resulted in an increase in stomatal conductance compared to the control.

### Total chlorophyll content

Total chlorophyll content significantly was affected by the interaction of light and nano-chelated potassium treatments. Light and nano-chelated potassium treatment increased the total chlorophyll content. According to Fig. 3b, it was found that the application of 3 g/l nano-chelated potassium in presence of blue light resulted in highest total chlorophyll content (3.95 mg/g FW). Although there was no statistically significant difference between recent treatment and concentration of 1.5 g/l nano-chelated potassium combined with red light. The lowest amount of total chlorophyll content (1 mg/g FW) was observed in the control.

### Photosynthesis rate

The findings of this research showed that both red and blue light combined with nano-chelated potassium leads to an increase in photosynthesis rate. Blue and red light treatments and concentrations of 1.5 and 3 g/l nano-chelated potassium caused an increase in photosynthesis rate up to 8.44 µmol CO_2_ m^−2^ s^−1^, which shows a notable increase in compared to the control (2.05 µmol CO_2_ m^−2^ s^−1^) (Fig. 3c).

### Leaf protein content

It was found that the combined treatment of light and nano-chelated potassium led to an increase in total protein content. As shown in Fig. 3d, increasing the concentration of nano-chelated potassium and light treatment caused an increasing trend in total protein content. Red light treatment and concentration of 3 g/l nano-chelated potassium gave the highest amount of total protein content (9.72 mg/g FW). The lowest amount of protein (3.4 mg/g FW) was observed in the control. According to Fig. 3d, it was found that the red light treatment and a concentration of 3 g/l nano-chelated potassium resulted in an almost threefold increase in total protein compared to the control.

### Carbohydrate content

Comparison of data means showed that red light treatment increased the amount of soluble carbohydrates and the plants exposed to red light treatment had the highest amount of soluble carbohydrates (686.22 mg/g FW). The lowest amount of soluble carbohydrates (485.77 mg/g FW) was observed in blue light treatment (Fig. 3e). According to Fig. 3f, it was found that nano-chelated potassium treatment increased the amount of soluble carbohydrates. Concentration of 3 g/l nano-chelated potassium had the greatest effect on increasing the amount of soluble carbohydrates, compared to the control.

### Petal anthocyanin content

The results of ANOVA showed that petal anthocyanin content was affected significantly by the interaction of light and nano-chelated potassium treatments (Table 2). Combined application of light and nano-chelated potassium led to an increase in petal anthocyanin content. The highest amount of petal anthocyanin content (18.60 mg/g FW) was earned with application of 3 g/l nano-chelated potassium in the presence of red light. The lowest amount of petal anthocyanin (9.93 mg/g FW) was observed in the control treatment (Fig. 3g).

### Petal phenolic content

Based on our findings, red light in combination with 3 g/l nano-chelated potassium had the greatest effect on the petal phenolic content increasing that was 140.92 mg gallic acid/g FW, which shows an increase in petal total phenolics compared to the control (Table 3).

**Table 3:** The biochemical traits and vase life of cut Dolce Vita rose in response to light and nano-chelated potassium treatments.

Treatments		Biochemical traits				
Light	Nano chelated potassium (g/L)	Phenolic content (mg gallic acid/g FW)	Flavonoids of leaves (mg/g FW)	Flavonoids of petals (mg/g FW)	Antioxidant capacity (%)	Flower vase life (days)
Control	0	57.063 ± 0.99h	13.280 ± 0.29h	6.306 ± 0.22d	51.699 ± 1.34g	13.66 ± 0.33f
	1.5	65.007 ± 1.33g	14.217 ± 0.15gh	6.983 ± 0.30cd	60.637 ± 1.09f	16.33 ± 0.33e
	3	69.263 ± 0.49f	15.310 ± 0.27fg	7.580 ± 0.10cd	66.413 ± 0.76e	17.33 ± 0.66de
Blue	0	89.453 ± 0.64e	16.533 ± 0.12ef	7.976 ± 0.22bc	70.633 ± 1.02d	18.66 ± 0.33d
	1.5	105.467 ± 1.17d	17.016 ± 0.16de	8.080 ± 0.12bc	76.590 ± 0.76c	20.66 ± 0.33c
	3	122.040 ± 0.93c	18.230 ± 0.38d	8.206 ± 0.56bc	80.080 ± 0.94bc	22.33 ± 0.33b
Red	0	132.507 ± 0.94b	20.543 ± 0.46c	8.373 ± 0.19bc	83.441 ± 0.39b	20.33 ± 0.33c
	1.5	138.620 ± 0.51a	23.2667 ± 0.53b	9.236 ± 0.49b	87.423 ± 0.50a	22.66 ± 0.33b
	3	140.920 ± 0.92a	25.153 ± 0.32a	11.346 ± 0.24a	89.697 ± 0.46a	26.66 ± 0.33a

### Flavonoids of leaves and petals

Results showed that the interaction of light and nano-chelated potassium treatments led to an increase in leaf and petals flavonoids. As shown in Table 3, the highest amount of leaf and petals flavonoids was gained in presence of red light and the concentration of 3 g/l nano-chelated potassium and the lowest amount was observed in the control treatment.

### Antioxidant capacity

Light in combination with nano-chelated potassium treatments affect antioxidant capacity. Red light treatment combined with the concentrations of 1.5 and 3 g/l nano-chelated potassium had the greatest effect on increasing the antioxidant capacity (Table 3).

### Petal H_2_O_2_ content

According to the results of ANOVA, the effect of light treatment on hydrogen peroxide content of petals was significant at the probability level of 1% (Table 2). Comparison of the means showed that both red and blue light treatment reduced the amount of H_2_O_2_ and the lowest amount of hydrogen peroxide (0.141 μmol/g FW) was observed in plants treated with red light (Fig. 3h).

### Flower vase life

Our findings showed that the combined use of light treatment and nano-chelated potassium led to an increase in flower vase life. As Table 3 shows, the highest vase life (26 days) was observed in the treatment of red light in combination with the concentration of 3 g/l nano-chelated potassium. The lowest vase life (13.66 days) was observed in the control treatment.

## Discussion

### Biomass & morphophysiological traits

Based on our findings, as seen in Fig. 2a–d, the use of nano-chelated potassium and red light led to an increase in the height of the flower stem, as well as an increase in its fresh and dry weight and the leaf area. In confirmation of the present research, in the study conducted by Poudel et al.^27^, the length of stem and internode and the number of leaves of grape seedlings grown in red LED light were higher compared to those grown at blue LED^28^.

In the present study, as seen in Fig. 3a, red light treatment led to an increase in stomatal conductance. Zeaxanthin is one of the components of the xanthophyll cycle in chloroplast, increases in the light, and decreases in the dark. On the other hand, red and blue light reduces the acidity of the lumen and therefore increases the content of zeaxanthin^29^; that is, it stimulates stomatal opening, resulting in more stomatal conductance, while darkness and green light prevents stomatal opening^29,30^. Potassium plays a crucial role in turgor regulation within the guard cells during stomatal movement^31^. As a rapid transport of K^+^ from the guard cells into the leaf apoplast resulted in stomatal closure, it is reasonable to think that K-deficient condition may disturb stomata opening. The inductive effect of K^+^ deficiency on stomatal closure and inhibited photosynthesis has been reported in several crop plants^32,33^.

Height increase due to the presence of blue light in some plants such as chrysanthemum (*Chrysanthemum morifolium*)^34^, parsley (*Petroselinum crispum*)^35^, petunia (*Petunia* × *hybrida*)^36^, and height reduction in plants such as Arabidopsis^37^, and poinsettia (*Euphorbia pulcherrima*)^38^ has been reported which shows different plants react differently to the blue light spectra. Blue light affects the expression and function of some genes responsible for gibberellin metabolism (such as *GA20ox*), and in some plant species, it leads to the creation of a signal by cryptochrome pigments and the reduction of gibberellin production, resulting in the reduction of stem height. While in some other species, the increase in the production of gibberellin and the increase in height occur in this process^39,40^. It has been reported that there is a close relationship between potassium and the growth of meristem tissues, and the strengthening effect of this element on plant growth regulators biosynthesis such as gibberellin and auxin which leads to the increased cell length and therefore, longitudinal growth of plant organs^41^. In a similar study, AM El-Naggar and B El-Nasharty^42^ reported that the use of foliar spraying of 2% potassium along with the soil application of 100% potassium had the most significant effects in plant height, number of leaves per plant, fresh and dry weight of leaves, number of flowers per spike, floret diameter, spike length, fresh and dry weight of florets, new corm diameter, fresh weight, and number of corms per plant in gladiolus.

During various studies, it has been reported that the wavelength range of 640–670 nm (red light) was effective in promoting photosynthetic activity, plant biomass and leaf growth. It also plays a substantial role in the development of the photosynthetic apparatus, the net rate of photosynthesis and primary metabolism^43,44^.

Opening of stomata occurs in response to low concentrations of internal carbon dioxide, high light intensity and high humidity^45^. In low availability of potassium, plants are more susceptible to wilting, and mild potassium deficiency affects photosynthesis rate by reducing stomatal conductance and photosynthetic biochemical reactions^46^. The application of 2.5 g/l nanopotassium fertilizer led to an increase in the stomatal conductance and stem diameter in pumpkin plants (*Cucurbita pepo*)^47^. In another study, using of potassium sulfate led to an increase in the fresh and dry weight, plant height, root length and leaf area of the Indian mustard (*Brassica juncea* L.)^48^.

Potassium is a macro-nutrient, which plays a crucial role in many plant physiological processes such as in cell turgidity and expansion, osmoregulation, stomatal opening and closure, and also works as an activator for several enzymes^49^. It seems that in this way, it has led to an increase in stomatal conductance in the present study. Similar to the present study, in a research, red LED light increased fresh and dry weight of aerial parts, number of stems, leaves and stem height in lemon balm (*Melissa officinalis*) seedlings compared to blue LED^50^.

Our findings are in confirmation with the results of Ouzounis et al.^51^ reported that the total biomass and height of roses was increased with a higher ratio of red to blue light. Findings show that the reaction of plants to specific light spectra’s or their different ratios is dependent on species or cultivars. The improving effects of blue and red lights in increasing the growth of plants such as lettuce (*Lactuca sativa*), radish (*Raphanus sativus*) and spinach (*Spinacia oleracea*)^52,53^, petunia^54^ and Norway spruce (*Picea abies*)^55^ has been reported. A group of researchers believe that the presence of low intensities of blue light can enhance the activity of pigments such as phototropins and as a result increasing the growrth of plants, but on the other hand, due to the different effects of blue light on other photoreceptors such as cryptochrome and phytochromes, it is possible that the response and reaction of different plant species to growth may different from each other^56,57^. Red light is received by phytochrome and affects the productive biomass and elongation of plants^58^. Blue light also affects photomorphological responses (such as adjusting leaf area and appearance) through phototropins and cryptochromes that act independently or synergistically with phytochrome^59,60^.

### Biochemical & photosynthetic traits

The presence of carbohydrates is necessary for flowers development and opening. Carbohydrates causes more water absorption, which increases the cell turgor and the freshness of petals, and therefore, the increase in flowers diameter is expectable^61^. In the present study, the interaction of light and nano-chelated potassium treatment led to an increase in content of photosynthetic pigments (Fig. 3c), and carbohydrates accumulation. Considering the role of carbohydrates in increasing water absorption, it can be expected that the fresh weight of the flower stems will increase.

Chlorophyll and carotenoids, by participation in the light trapping complex, plays a very important role in the efficiency of photosynthesis, light absorption and electron transfer^62^. According to the research conducted by Massa et al.^63^ and Niakan et al.^64^, the improving effect of light on growth and chlorophyll content has been emphasized. Red and white lights affects the production of chlorophyll precursors, and increased chlorophyll biosynthesis. Red light irradiation alone was unsuccessful for chlorophyll biosynthesis, while the combination of blue and red light irradiation was necessary for this process and improved chlorophyll biosynthesis and thus photosynthesis rate^65^. The red light of the visible spectrum is needed for the growth of the photosynthetic apparatus and photosynthesis, while the blue light is needed for the synthesis of chlorophyll, chloroplast, stomata opening, and photomorphogenesis^66^. Increasing the amount of photosynthesis in the present study due to red light treatment is likely related to the development of the photosynthetic apparatus. Red light in combination with blue light are best for photosynthesis^10^. Blue light affects stomata width and stomatal conductivity, plant height, and chlorophyll biosynthesis^67^. In confirmation of the results of the present study, Chung et al.^68^ reported an increase in the content of chlorophyll in the orchid (*Oncidium* ‘Gower Ramsey) plants exposed to combined red and blue light.

In this study, the use of nano-chelated potassium led to an increase in photosynthetic pigments. Potassium does not have a direct effect on the activity of rubisco carboxylase enzyme, but by increasing the synthesis of carboxylation enzymes such as phosphoenolpyruvate carboxylase (PEP Case), it stimulates the stabilization of carbon dioxide and increases photosynthesis rate^69^. It has been reported that the use of different concentrations of nano-chelated potassium can lead to a significant increase in the amount of chlorophyll a and b, the amount of soluble sugars and protein content in the leaves of plants^70^. In confirmation of the results of the present study, AM El-Naggar and B El-Nasharty^42^ reported an increase in the content of chlorophyll a and b in the leaves of the gladiolus plant in response to potassium application.

### Anthocyanin, protein, phenolics & flavonoids

According to Fig. 3e–f, red light and nano-chelated potassium treatment increased the amount of soluble carbohydrates. Also the amount of total protein and anthocyanin of flowers were affected by the interaction of light and nano-chelated potassium (Fig. 3d,g). Light through the activation of some genes (*CHS, CHI* & *F3H*) involved in anthocyanin biosynthesis, causes the accumulation of anthocyanin in green tissues and cultured cells^71^. Researches shows that along with monospectral red light, the combination of blue and red LED spectrum also causes the accumulation of primary metabolites as well as the increase of anthocyanins, polyphenols and flavonoids^72,73^. However, red LEDs have a greater effect on anthocyanin accumulation than blue LEDs. This issue may was attributed to the increased expression of anthocyanin biosynthesis genes (for example, *MdMYB10* and *MdUFGT*) due to red light treatment^74^. It has been demonstrated that potassium increases the production of starch and carbohydrates^75^.Chalcone isomerase, one of the key enzymes of anthocyanin biosynthesis can activated by a signal from soluble carbohydrates, so the accumulation of carbohydrates can enhance the production of anthocyanins^76^. Results from a research on *Prunus* × *yedoensis* 'Somei-yoshino' petals coloring, showed that a combination of blue and red light causes more anthocyanin production than monochromatic blue light, which indicates the correlation between blue and red light receptors^77^.

Findings shown that potassium plays an essential role in the construction of polymer compounds in plants. In plants that suffer from potassium deficiency, simple sugars, soluble nitrogen compounds and amino acids are accumulated and the amount of starch and leaf protein is reduced^78^. Potassium participates in the last stage of the protein production process. K^+^ is an activator of dozens of important enzymes, and contributes in protein synthesis, sugar transport, N and C metabolism, and photosynthesis. It plays an important role in crop yield and quality improvement^31,79–83^. Therefore, it can be assumed that the using of nano-chelated potassium may be a reason for the increase in the protein content.

In the present study, the application of red light and nano-chelated potassium led to an increase in the antioxidant capacity and the amount of phenolic compounds and flavonoids of petals. A direct relationship has been reported between the antioxidant properties and the amount of phenolic compounds and anthocyanin content in bayberry (*Myrica pensylvanica*)^84^ and blueberries (*Vaccinium corymbosum*)^85^. Light quality has a significant effect on the accumulation of different metabolites in plants^86^. Flavonoids are involved in many aspects of plant growth and development, including resistance to pathogens, pigment production, protection against UV rays, pollen development, and seed coat development^87^. The increase in the amount of total phenolic compounds due to potassium treatment can be attributed to the increase in the expression of genes involved in the biosynthesis of phenylpropanoids, and especially the increase in the expression of the gene responsible for the biosynthesis of the enzyme phenyl-alanine-ammonialyase (PAL), which is the first enzyme in the phenolic compound’s synthesis pathway^88^.

### H_2_O_2_ and vase life

As seen in Fig. 3h, the interaction of light and nano-chelated potassium led to a two-time increase in the vase life of cut roses. Aging in plants is an oxidative process and includes biochemical, physiological, hormonal and structural changes, which causes the destruction of large molecules such as protein, nucleic acids and lipids^89^. One of the reasons for senescence in plant tissues is the involvement of reactive oxygen species (ROS) including hydrogen peroxide and hydroxyl, which cause the production of free radicals and the destruction of proteins, lipids and nucleic acid, and finally flower senescence^90,91^. Red light increases the total phenol content as well as radical scavenging capacity^92^ that slows down the flower aging. Potassium is an essential macronutrient that contributes in many physiological aspects related to osmotic adjustment, maintaining turgor pressure, cell expansion, plasma membrane electric potential balancing, and homeostatic regulation of pH^49^. In the present study, the application of red light and nano-chelated potassium led to the improving of some indices related to flower quality and vase life such as photosynthetic pigments content, antioxidant capacity and phenolic compounds, so it can be expected that the vase life of rose flowers will increase due to the treatment of red light and nano-chelated potassium.

## Conclusion

**Figure 4:**
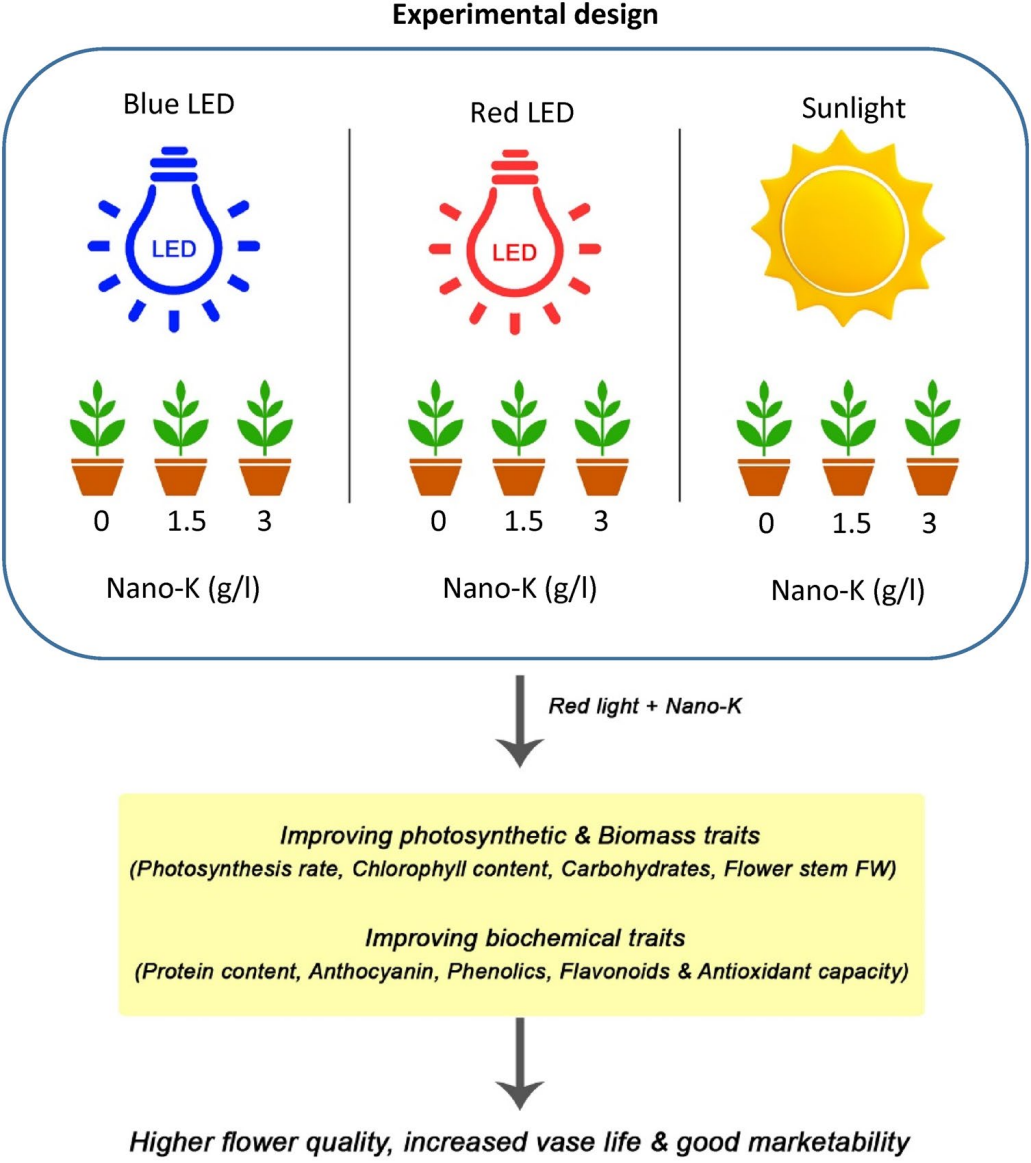
A schematic work model of experimental design and findings.

Using of LED lighting and nano-chelated potassium improved morphological and biochemical traits of ‘Dolce Vita’ rose flower. Red light in combination with nano-chelated potassium increased the photosynthesis rate and biomass of rose plants (Fig. 4). The biochemical traits such as protein content, antioxidant content, total phenolics and flavonoids, also was affected by red light and nano-chelated potassium that led to color enhancement in petals edge and higher marketability values. In addition, the flower vase life was increased notably. In general, it can be concluded that the use of red light and nano-chelated potassium, can be effective in improving the quality of the produced flowers, especially in seasons that plants is experiencing a decrease in light intensity and duration.

## Data Availability

The datasets used and/or analysed during the current study available from the corresponding author on reasonable request.
